# Burnout and intent to stay among nurses in a private tertiary hospital

**DOI:** 10.1192/j.eurpsy.2025.1648

**Published:** 2025-08-26

**Authors:** C. I. C. Uy

**Affiliations:** 1 Department of Psychiatry, The Medical City, Pasig, Philippines

## Abstract

**Introduction:**

Burnout is an occupational phenomenon that may be a risk factor for several mental health disorders. It is defined by three dimensions namely emotional exhaustion (EE), depersonalization (DP), and personal accomplishment (PA). The nursing workforce experiences high levels of burnout taking a toll not only on their mental state but also their intent to stay leading to issues on shortage.

**Objectives:**

This study aims to assess association of burnout and its dimensions to intent to stay of medical nurses working in a private tertiary hospital in the Philippines.

**Methods:**

Analytical cross-sectional study using secondary data conducted by the Nursing Services Group of the private tertiary hospital last March 2023. A survey was done on nurses’ perceptions of their working condition using Maslach Burnout Inventory and McCain’s Intent to Stay tools. Variables were assessed through simple and multiple linear regression analyses.

**Results:**

On simple linear regression, burnout, civil status, years of experience, and years of tenure revealed significance in their respective categories. EE and DP dimensions showed negative association to intent to stay. On multiple linear regression, only burnout (p< 0.000 and -0.028) and those married with children (p< 0.000 and -0.028) had significant association. EE consistently showed negative association however, DP and PA had positive association to intent to stay. In most literatures, DP is associated to lower intent to stay as it is equated to cynicism or detachment in interpersonal relations which can manifest as negative or inappropriate attitudes towards clients, irritability, loss of idealism, and withdrawal (Maslach et al. World Psychiatry 2016; 15 103-11). However, in this study, nurses who were more detached had surprisingly higher intent to stay which may show how cynicism can be protective. It is a cognitive method of creating a protective distance to prevent them from letting their job performance suffer especially when dealing with the physical and emotional exhaustion, and feelings of ineffectiveness caused by excessive and prolonged stress (Akyurt et al. Medical Science and Discover 2023; 10 918-928).

**Image 1:**

**Figure:**
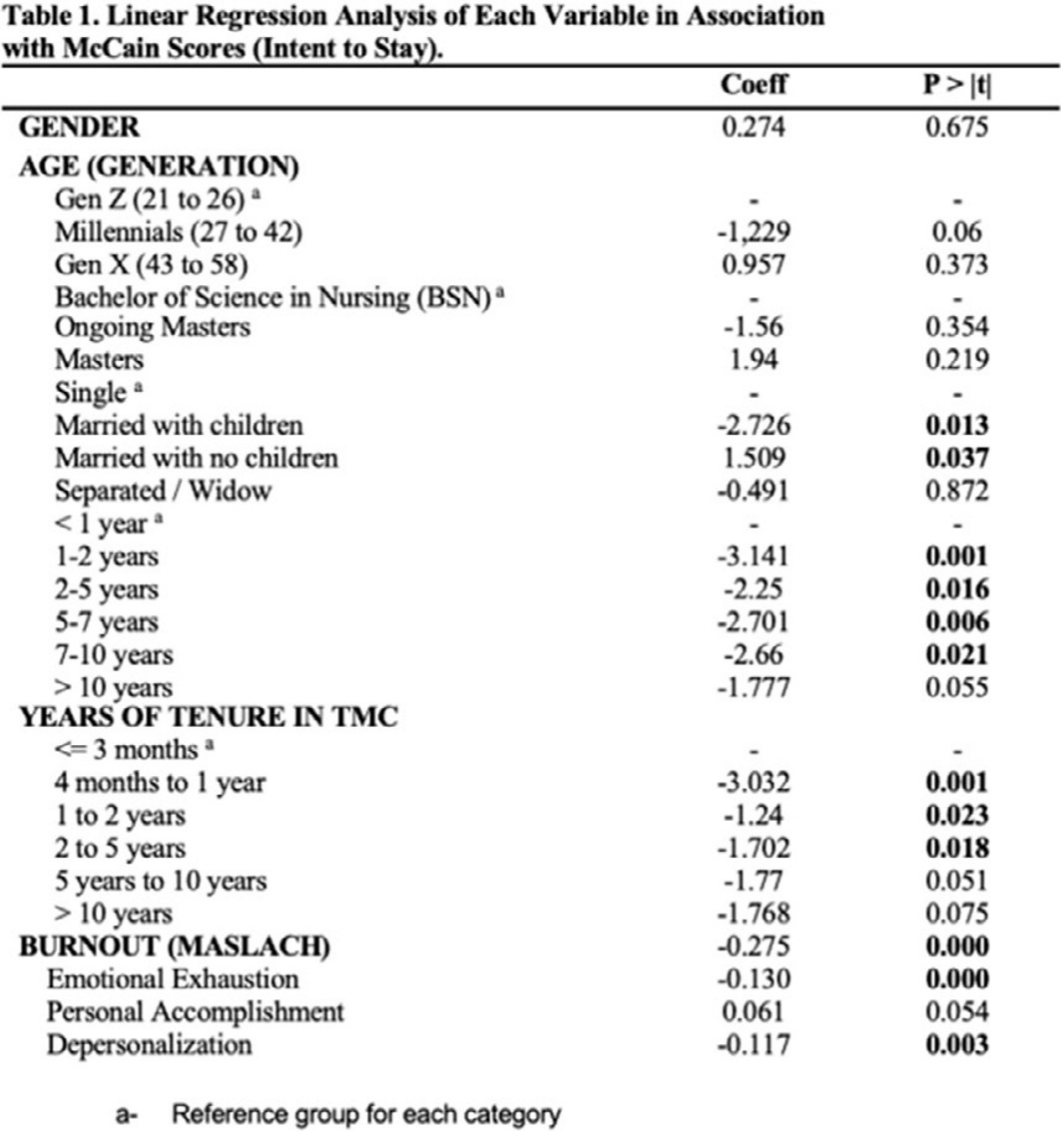

**Image 2:**

**Figure:**
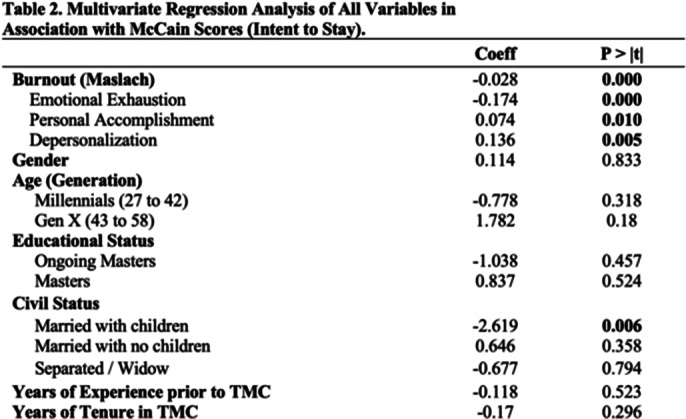

**Conclusions:**

Consistent with global studies, burnout is associated to lower intent to stay among nurses. However, it is beneficial to have more research looking further into the comprehensive role of cynicism in burnout. In this study, nurses have built some level of cynicism that is able to preserve themselves in negative situations. However, with no proper management, depersonalization can aggravate ultimately leading to feelings of inadequacy and lower intent to stay. A deeper and more contextualized understanding about this phenomenon may help administrators improve existing operations and policies that can help foster a healthier working environment for the nurses.

**Disclosure of Interest:**

None Declared

